# Predictors of nirmatrelvir–ritonavir receipt among COVID-19 patients in a large US health system

**DOI:** 10.1038/s41598-024-57633-7

**Published:** 2024-03-29

**Authors:** Deborah E. Malden, John M. McLaughlin, Vennis Hong, Joseph Lewnard, Bradley K. Ackerson, Laura Puzniak, Jeniffer S. Kim, Harpreet Takhar, Timothy B. Frankland, Jeff M. Slezak, Sara Y. Tartof

**Affiliations:** 1 Department of Research and Evaluation, Kaiser Permanente Southern California, 100 South Los Robles, Pasadena, CA 91101 USA; 2 Pfizer Inc, New York, USA; 3 Division of Epidemiology, School of Public Health, University of California, Berkeley, Berkeley, CA 94720 USA; 4 Division of Infectious Diseases and Vaccinology, School of Public Health, University of California, Berkeley, Berkeley, CA 94720 USA; 5 Center for Computational Biology, College of Engineering, University of California, Berkeley, Berkeley, CA 94720 USA; 6 Department of Health Systems Science, Kaiser Permanente Bernard J. Tyson School of Medicine, Pasadena, CA 91101 USA

**Keywords:** Medical research, Epidemiology, Health services, Public health, Therapeutics

## Abstract

A clear understanding of real-world uptake of nirmatrelvir–ritonavir for treatment of SARS-CoV-2 can inform treatment allocation strategies and improve interpretation of effectiveness studies. We used data from a large US healthcare system to describe nirmatrelvir–ritonavir dispenses among all SARS-CoV-2 positive patients aged ≥ 12 years meeting recommended National Institutes of Health treatment eligibility criteria for the study period between 1 January and 31 December, 2022. Overall, 10.9% (N = 34,791/319,900) of treatment eligible patients with SARS-CoV-2 infections received nirmatrelvir–ritonavir over the study period. Although uptake of nirmatrelvir–ritonavir increased over time, by the end of 2022, less than a quarter of treatment eligible patients with SARS-CoV-2 infections had received nirmatrelvir–ritonavir. Across patient demographics, treatment was generally consistent with tiered treatment guidelines, with dispenses concentrated among patients aged ≥ 65 years (14,706/63,921; 23.0%), and with multiple comorbidities (10,989/54,431; 20.1%). However, neighborhoods of lower socioeconomic status (upper third of neighborhood deprivation index [NDI]) had between 12% (95% CI: 7–18%) and 28% (25–32%) lower odds of treatment dispense over the time periods studied compared to the lower third of NDI distribution, even after accounting for demographic and clinical characteristics. A limited chart review (N = 40) confirmed that in some cases a decision not to treat was appropriate and aligned with national guidelines to use clinical judgement on a case-by-case basis. There is a need to enhance patient and provider awareness on the availability and benefits of nirmatrelvir–ritonavir for the treatment of COVID-19 illness.

## Introduction

Nirmatrelvir is an oral antiviral that, when co-administered with ritonavir within 5 days of symptom onset, is highly effective at reducing the risk of hospitalization and death among patients with mild-to-moderate COVID-19 who are at risk for progression to severe disease^1–6^. Accordingly, nirmatrelvir–ritonavir received Emergency Use Authorization (EUA) from the Food and Drug Administration (FDA) in December 2021. Nirmatrelvir–ritonavir treatment eligibility depends on the presence of underlying risk factors for progression to severe COVID-19 including age and vaccination status, weight, renal and hepatic function, and current use of select medications known to interact with nirmatrelvir–ritonavir. In the United States, initial guidelines recommended a tiered prioritization approach to treatment based on clinical risk^7^. As knowledge and treatment availability expanded throughout 2022, recommendations adapted to widen treatment eligibility criteria, eventually including all persons aged 65 years and older or aged 12 years and older with one or more clinical risk factors for progression to severe COVID-19. More recently (i.e., after our study period), treatment eligibility criteria have expanded further to include all adults aged 50 years and older. A clear understanding of the population-level distribution of treatment allocation has important implications for the study design and interpretation of real-world effectiveness studies, as well as informing strategies for improved treatment access.

Despite evidence that underrepresented minority populations have a higher risk of severe COVID-19 outcomes^8^, national surveillance data suggests that disparities among socio-economic characteristics existed during early treatment allocation of nirmatrelvir–ritonavir^9^. As well as exacerbating disparities in health outcomes, such disparities in treatment allocation have the potential to cause bias in real-world effectiveness studies since nirmatrelvir–ritonavir recipients and non-recipients will exhibit different general healthcare seeking behaviors which must be defined and accounted for in the interpretation of real-world analysis. However, it is challenging to draw clear conclusions about disparities in treatment allocation because socio-economic characteristics are closely correlated with clinical and demographic factors which themselves determine treatment eligibility.

Therefore, to better understand nirmatrelvir–ritonavir uptake, a robust analysis using complete individual-level electronic health record (EHR) data from a large and diverse population is needed. While several real-world EHR-based studies have assessed the effectiveness of nirmatrelvir–ritonavir against severe disease^1,2,4–6,10,11^, few describe temporal variation in treatment patterns across clinical and demographic characteristics. Furthermore, most prior studies covered a brief period of the first few months of 2022, and treatment patterns have likely changed given the more recent expanded availability of nirmatrelvir–ritonavir.

We address this gap by describing nirmatrelvir–ritonavir dispense patterns across population characteristics within a large and diverse US integrated healthcare system during January-December 2022.

## Methods

### Data sources

We conducted a retrospective cohort study using EHR data from Kaiser Permanente Southern California (KPSC). KPSC is an integrated healthcare system that provides care to more than 4.6 million members whose socio-demographics approximately mirror the diverse population of Southern California^12^. Comprehensive EHRs used for this study included information on demographics, diagnoses, pharmacy dispenses, laboratory tests, and vaccinations. Nirmatrelvir–ritonavir was available at no cost to recipients in the U.S. through the Department of Health and Human Services as of December 23, 2021. Out-of-network dispenses for nirmatrelvir–ritonavir were captured through insurance claims reimbursements, although these are thought to occur infrequently due to the ease of nirmatrelvir–ritonavir access within-network. The study was approved by Kaiser Permanente Southern California’s Institutional Review Board, with a waiver for informed consent. All study methods were carried out in accordance with US guidelines and regulations.

### Study population

The primary study population was comprised of patients aged at least 12 years with EHR documentation of a positive SARS-CoV-2 test (i.e., documented positive PCR or antigen test across all healthcare settings or self-reported positive test for SARS-CoV-2) and identified as eligible for treatment with nirmatrelvir–ritonavir between 1 January and 31 December 2022. To ensure complete capture of comorbidities, medication use, and healthcare utilization, we excluded patients without continuous membership for at least 1 year prior to treatment dispense (allowing for a 45-day gap to account for potential delays in membership renewal). Due to the known clustering of clinical, demographic, and socio-economic factors with nirmatrelvir–ritonavir treatment eligibility, only treatment eligible patients were included in the main analysis, defined according to the US National Institutes of Health (NIH) COVID-19 Treatment Guidelines which were the consistent KPSC treatment guidelines provided to KPSC clinicians over the study period^7^. In brief, patients with documentation of positive SARS-CoV-2 tests and symptoms consistent with COVID-19 were identified as treatment eligible if they weighed at least 40 kg and were aged ≥ 12 years with an underlying risk factor for severe COVID-19 illness in the year prior to the date of SARS-COV-2 test (Appendix A)^13^. Patients were excluded from the treatment eligible cohort if they had documentation of severe renal impairment (eGFR < 30 mL/min) or severe liver impairment within 1 year prior to the date of SARS-CoV-2 test. Patients were also excluded if they had received contraindicated medications within 6 months prior to SARS-CoV-2 infection^14,15^, received molnupiravir within one calendar day of diagnosis, or if they were hospitalized at the time of COVID-19 diagnosis. The list of contraindicated medications changed as knowledge of potential drug interactions evolved over time (Appendix B).

### Covariates

Treatment patterns were described across several selected covariates of interest, including age, sex, race/ethnicity (non-Hispanic White, non-Hispanic Black, non-Hispanic Asian; Hispanic; or other/unknown), body mass index (BMI), neighborhood deprivation index (NDI), COVID-19 vaccination status, prior documented SARS-CoV-2 infection, health insurance status (Medicaid, Medicare, Commercial, Other), selected common chronic comorbidities (Appendix C) and healthcare utilization in the year prior (outpatient, Emergency Department [ED], and inpatient encounters). NDI is a composite measure of socioeconomic vulnerability derived from a number of census-tract level characteristics^16^. Cut points for NDI were derived from the continuous measure across all census tracts of all KPSC members, with higher values representing greater levels of community deprivation. The number of comorbid conditions was calculated as the sum of specific high-risk conditions, including obesity and immunocompromised status, as defined by CDC^13^. Vaccination status was categorized by the number of vaccinations (0, 1, 2, or ≥ 3) administered at least 14 days prior to the observed SARS-CoV-2 positive test date or dispense date where there was no record of a positive SARS-COV-2 test. Prior SARS-CoV-2 infections were defined as documentation of SARS-CoV-2 infection more than 90 days prior to the date of SARS-CoV-2 positive test or dispense date occurring during the study period.

Symptoms associated with SARS-CoV-2 infection were defined as fever, cough, chills, dyspnea, sore throat, anosmia, myalgia, abdominal pain, diarrhea, vomiting/nausea, fatigue, or headache occurring within 14 days before or after the SARS-CoV-2 test date. These symptoms were extracted from three sources: (1) structured questionnaires administered at the time of SARS-CoV-2 test; (2) diagnoses codes; or (3) from unstructured text fields within EHRs using natural language processing, as described elsewhere^17^.

### Statistical analysis

Descriptive analyses of patient characteristics were presented as counts/frequencies for categorical variables and mean (SD) or median (IQR) for continuous variables. Logistic regression was used to estimate adjusted odds ratios (aOR) and 95% confidence intervals (CIs) for treatment with nirmatrelvir–ritonavir among the treatment eligible cohort across equal thirds of NDI, stratified by date of SARS-CoV-2 test (Jan 1–Mar 31; Apr 1–Jun 30; Jul 1–Sep 30; Oct 1–Dec 31, 2022). Models were adjusted for age, sex, race/ethnicity, comorbidities in the year prior to the positive SARS-CoV-2 test date (Charlson weighted comorbidity index^18^), health insurance status (Medicaid, Medicare, commercial, or other), healthcare utilization in the year prior to the positive SARS-CoV-2 test date and COVID-19 vaccination status (0, 1, 2, or ≥ 3 doses). All analyses were performed using SAS (version 9.4; SAS Institute) and graphical visualizations were produced in R (version 4.2.2).

### Physician chart reviews

A subset of 40 patients who met the treatment eligibility criteria but did not receive treatment with nirmatrelvir–ritonavir were selected for a physician chart review. Since NDI was strongly associated with treatment dispense, samples were randomly selected within equal thirds of the NDI distribution. Chart reviews were conducted by a trained co-investigator (BKA) and the findings were discussed and interpreted by the wider study team. The purpose of the physician chart review was to identify reasons underlying lack of treatment dispenses, with an overall objective to identify potential barriers to treatment.

### Sensitivity analysis

EHR data may be insufficient to identify all patients who were nirmatrelvir–ritonavir treatment-eligible or ultimately received treatment. Moreover, requiring EHR documentation of a positive SARS-CoV-2 test likely underestimated the total number of true infections for the KPSC population (and thus overall use of nirmatrelvir–ritonavir). To understand the impact of these two potential limitations, in addition to the primary analysis, we evaluated nirmatrelvir–ritonavir uptake among (1) all individuals with a documented positive SARS-CoV-2 test over the study period, regardless of documented treatment eligibility and (2) the overall population regardless of whether there was evidence of a positive SARS-CoV-2 test in the EHR.

## Results

**Figure 1:**
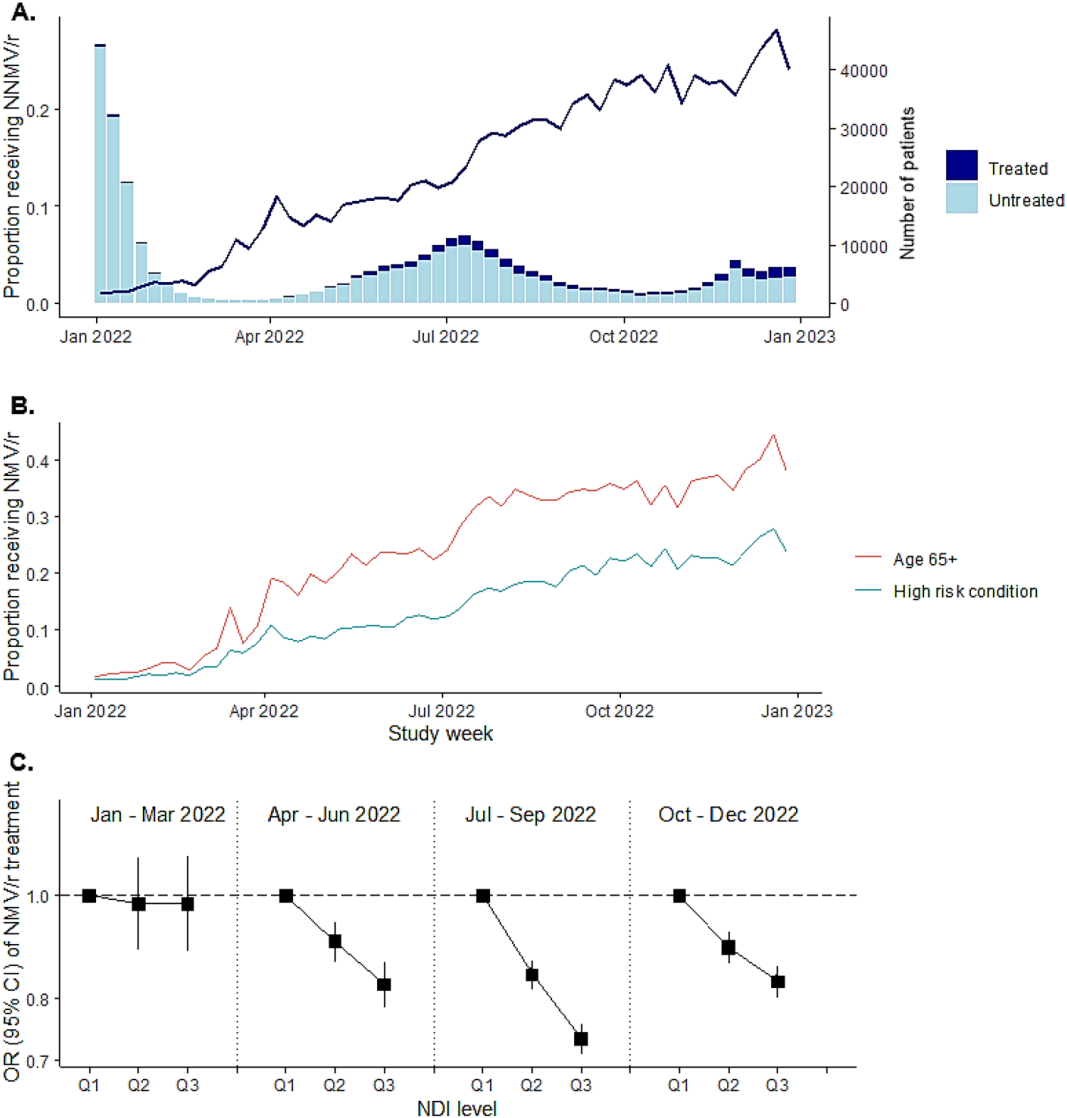
Proportion of treatment eligible SARS-CoV-2 positive patients receiving nirmatrelvir–ritonavir treatment dispense over time, by clinical risk factor and neighborhood deprivation index (NDI). (**A**) Proportion of patients with a confirmed SARS-CoV-2 infection and documentation of at least one treatment eligibility-criteria receiving a nirmatrelvir–ritonavir treatment dispense, by time period of infection; (**B**) Proportion of patients with a confirmed SARS-CoV-2 infection and at least one high risk condition (defined as an underlying risk factor for severe COVID-19 illness in the year prior to the date of SARS-COV-2 test [Appendix A]) or aged ≥ 65 years receiving a nirmatrelvir–ritonavir treatment dispense, by time of infection; (**C**) Odds Ratio (95% CI) of treatment dispense among patients with a confirmed SARS-CoV-2 infection and documentation of at least one treatment eligibility-criteria, by time period of infection and NDI. Q1 of NDI is the reference across all time periods, respectively. *NDI* Neighborhood Deprivation Index, *OR* Odds Ratio, *Q1* Tertile 1, *Q2* Tertile 2, *Q3* Tertile 3.

A total of 319,900 patients with SARS-CoV-2 infections were identified as treatment-eligible according to NIH guidelines and met the study inclusion criteria (Appendix D). Mean age was 47.8 (SD 17.7) years, 62.4% (199,584/319,900) were female, 47.3% (151,397/319,900) were Hispanic, and 16.8% had type II diabetes (53,661/319,900). Overall, 62.8% of the treatment-eligible cohort had at least one underlying chronic medical condition associated with increased risk to progression to severe COVID-19 and 20.0% were aged ≥ 65 years (Appendix E). Among these patients, a total of 34,791 (10.9%) had at least one documented dispense for nirmatrelvir–ritonavir during the study period (Table 1). Overall, the weekly proportion of treatment eligible persons with SARS-CoV-2 infection receiving nirmatrelvir–ritonavir increased rapidly, from 1.1% in January 2022 to 24.0% in December 2022 (Fig. 1A), with the majority (> 75%) of dispenses occurring after July 1, 2022.

**Table 1:** Proportion of treatment eligible persons^a^ with SARS-CoV-2 infection receiving nirmatrelvir–ritonavir by selected baseline characteristic.

	All SARS-CoV-2 positive patients eligible to receive treatment^a^	
	Total, col %	Treatment dispensed, row %
Total	319,900	34,791 (10.9%)
Sex		
Men	120,316 (37.6%)	13,196 (11.0%)
Women	199,584 (62.4%)	21,595 (10.8%)
Age, years		
12–17	11,840 (3.7%)	125 (1.1%)
18–30	44,826 (14.0%)	1505 (3.4%)
31–44	89,250 (27.9%)	5522 (6.2%)
45–64	110,063 (34.4%)	12,933 (11.8%)
65+	63,921 (20.0%)	14,706 (23.0%)
Mean (SD)	47.8 (17.7)	58.9 (16.1)
Race/ethnicity		
Asian	34,532 (10.8%)	4643 (13.4%)
Black	25,552 (8.0%)	2624 (10.3%)
Hispanic	151,397 (47.3%)	12,833 (8.5%)
Other/unknown	15,352 (4.8%)	1288 (8.4%)
White	93,067 (29.1%)	13,403 (14.4%)
BMI^b^, kg/m^2^		
< 18.5	3283 (1.0%)	275 (8.4%)
18.5–24.9	59,610 (18.6%)	6772 (11.4%)
25–29.9	84,550 (26.4%)	9955 (11.8%)
30+	172,457 (53.9%)	17,789 (10.3%)
Insurance plan		
Medicaid	32,972 (10.3%)	3016 (9.1%)
Medicare	51,870 (16.2%)	12,272 (23.7%)
Commercial	219,157 (68.5%)	17,919 (8.2%)
Other	14,727 (4.6%)	1478 (10%)
Unknown	1174 (0.4%)	106 (9%)
NDI, quintiles		
Q1	65,025 (20.3%)	9302 (14.3%)
Q2	68,510 (21.4%)	8031 (11.7%)
Q3	67,206 (21.0%)	6866 (10.2%)
Q4	63,825 (20.0%)	5972 (9.4%)
Q5	55,271 (17.3%)	4612 (8.3%)
Unknown	63 (–)	8 (12.7%)
Comorbidities^b,c^		
0–1	265,469 (83.0%)	23,802 (9.0%)
2–3	36,551 (11.4%)	7290 (19.9%)
4+	17,880 (5.6%)	3699 (20.7%)
Comorbidities^b^		
Chronic kidney disease	14,360 (4.5%)	2838 (19.8%)
Coronary heart disease	1563 (0.5%)	256 (16.4%)
Diabetes (type II)	53,661 (16.8%)	9443 (17.6%)
Heart failure	5118 (1.6%)	917 (17.9%)
Stroke	3314 (1.0%)	596 (18%)
COPD	4631 (1.4%)	1058 (22.8%)
Total	319,900	34,791 (10.9%)
SARS-CoV-2 test type		
PCR test	232,426 (72.7%)	17,317 (7.5%)
Rapid antigen	25,190 (7.9%)	7326 (29.1%)
Self-reported	62,284 (19.5%)	10,148 (16.3%)
Annual healthcare visit^d^		
Outpatient visit	311,490 (97.4%)	34,316 (11%)
ED/Inpatient visit	75,346 (23.6%)	8817 (11.7%)
Virtual encounter	247,466 (77.4%)	27,931 (11.3%)
None	4570 (1.4%)	275 (6.0%)
COVID-19 vaccination doses received^e^		
0	41,808 (13.1%)	2155 (5.2%)
1	10,487 (3.3%)	586 (5.6%)
2	101,662 (31.8%)	4840 (4.8%)
3+	165,943 (51.9%)	27,210 (16.4%)
Time from last dose		
< 6 weeks	34,590 (10.8%)	4585 (13.3%)
6 weeks–3 months	15,644 (4.9%)	1788 (11.4%)
> 3 months	227,858 (71.2%)	26,263 (11.5%)
NA/unvaccinated	41,808 (13.1%)	2155 (5.2%)
Prior SARS-CoV-2 infection^f^		
Yes	38,531 (12.0%)	3139 (8.1%)
No	281,369 (88.0%)	31,652 (11.2%)
COVID-19 symptoms documented in EHR^g^		
Before diagnosis	270,218 (84.5%)	29,352 (10.9%)
Date of diagnosis	36,866 (11.5%)	4595 (12.5%)
After diagnosis	12,816 (4.0%)	844 (6.6%)

^a^Treatment eligibility defined according to the NIH COVID-19 Treatment Guidelines, including a documented SARS-CoV-2 positive test.

^b^Documented within the year prior to positive SARS-CoV-2 test.

^c^Co-morbidities defined according to ICD-10 codes included in Appendix A.

^d^Groupings are not mutually exclusive.

^e^Vaccination must have occurred at least 14 prior to the date of SARS-CoV-2 test. Doses received within 14 days of one another were considered duplicated records and the earliest dose was used as the vaccination date in the analysis.

^f^PCR or rapid test with a positive result prior to the SARS-CoV-2 test occurring over the study period.

^g^Symptoms were identified from structured and unstructured EHR data using an algorithm developed and validated within the KPSC population.

*NDI* Neighborhood Deprivation Index, *BMI* Body-Mass Index, *EHR* Electronic Health Records, *NA* Not applicable.

In line with NIH tiered treatment guidelines, nirmatrelvir–ritonavir dispenses were concentrated among persons aged ≥ 65 years (14,706/63,921; 23.0%) and among persons with at least two documented comorbidities (10,989/54,431; 20.1%). Treatment rates were also higher among patients who had received a third dose of COVID-19 vaccination (27,210/165,943; 16.4%) (Table 1). Treatment rates were also particularly high for White (13,403/93,067; 14.4%) and Asian patients (4643/34,532; 13.4%) compared with Hispanic (12,833/151,397; 8.5%), Black (2624/25,552; 10.3%) or other/unknown race/ethnicities (1288/15,352; 8.4%). The proportion of treatment eligible patients receiving nirmatrelvir–ritonavir over the study period appeared to decline at higher levels of community deprivation, from 14.3% in the lowest NDI quintile (i.e., most affluent) to 8.3% in the highest NDI quintile (i.e., most deprived). The proportion of treatment eligible persons patients aged ≥ 65 years and the proportion of patients with at least one treatment-qualifying high-risk condition receiving nirmatrelvir–ritonavir increased at similar rates by study week (Fig. 1B).

In adjusted analyses among treatment eligible patients, there was no difference in the likelihood of receiving treatment across thirds of the NDI distribution within the first 3 months of 2022 (Fig. 1C). However, for each 3-month interval following this period, residential areas within the highest third of the NDI distribution (i.e., more deprived) were associated with between 12% (95% CI: 7–18%) and 28% (95% CI: 25–32%) lower odds of treatment dispense compared to the lower third of the NDI distribution (i.e., more affluent). Although they persisted from Mar 2022 onwards, disparities appeared to widen during Jul–Sep 2022 and narrowed slightly during Oct–Dec 2022. These disparities were not explained by age, however there was some evidence of effect modification by race/ethnicity in the latter half of 2022, whereby White ethnicity was less associated with disparities in treatment dispense across thirds of NDI compared with non-White ethnicity (Appendix F).

In a sensitivity analysis of all KPSC patients with SARS-CoV-2 infections over the study period (including those not meeting the treatment eligibility criteria, N = 541,785), 8.2% (N = 44,645) received nirmatrelvir–ritonavir (Appendix G). Similar to the primary analyses, in general, treatment dispense aligned with recommendations, with higher dispense rates among patients who were aged ≥ 65 years (17,949/88,095; 20.4%), White (16,985/148,325; 11.5%), or with > 1 documented comorbidity (13,799/77,215; 17.9%). Patients residing in more affluent areas also had higher dispense rates compared to patient residing in more deprived areas (10.8% vs. 6.3% for NDI Quintile 1 vs. 5, respectively). Most SARS-CoV-2 positive patients were diagnosed via PCR test (75.7%). However, use of PCR tests decreased rapidly while at-home testing increased significantly during the later months of the study period (Appendix H). Overall, 41% of all nirmatrelvir–ritonavir recipients did not have a SARS-CoV-2 test documented in their EHR (Appendix I). Compared to nirmatrelvir–ritonavir recipients with documented SARS-CoV-2 infections, these recipients were older, had more chronic illness, and less healthcare use. In general, patients who were prescribed nirmatrelvir–ritonavir but failed to fill their prescription were younger, healthier, and unvaccinated compared to patients who filled their prescription (Appendix J).

### Physician chart reviews

Physician chart review was conducted for 40 patients meeting the treatment eligibility criteria but for whom there was no documentation of nirmatrelvir–ritonavir dispense (Appendix K). Of these, most of the treatment decisions appeared to be appropriate, with patients either presenting as asymptomatic by the time follow-up was conducted (N = 5; 13%) or presenting > 5 days after symptom onset, outside the recommended treatment window (N = 17; 43%). Ten of these patients appropriately received alternative treatments, including remdesivir or monoclonal antibody therapy. However, in more than a third of cases (N = 15; 38%), chart review confirmed patients’ eligibility for treatment according to NIH guidelines; for these patients, chart reviews were unable to confirm whether treatment was offered at the time of medical consultation or COVID-19 diagnosis.

## Discussion

In this real-world study using EHR data from over 310,000 treatment-eligible patients with SARS-CoV-2 receiving care from an integrated healthcare system, several important patterns in nirmatrelvir–ritonavir treatment dispense were identified. Uptake of nirmatrelvir–ritonavir was rapid following issuance of the EUA, particularly among older age groups, persons of White and Asian ethnicity, residents of low-vulnerability (i.e., less deprived) areas, and those with treatment-indicated high-risk conditions. However, despite more relaxed prescribing over time, by the end of 2022, only about one quarter of treatment eligible patients with SARS-CoV-2 infections were receiving nirmatrelvir–ritonavir. This demonstrates a need to enhance patient and provider awareness on the availability and benefits of nirmatrelvir–ritonavir for the treatment of COVID-19 illness. This study builds upon earlier reports by extending the observation period and leveraging the availability of comprehensive EHR data to assess real-world treatment dispense patterns over time and by multiple simultaneous clinical and demographic characteristics.

Consistent with other studies^1,2,5,6,10,11,19,20^, we identified a sharp rise in the proportion of patients with SARS-CoV-2 infections receiving nirmatrelvir–ritonavir over the study period, roughly coinciding with expanding treatment availability and waves of SARS-CoV-2 infections. In general, treatment allocation aligned with NIH tiered guidelines, with the majority of dispenses concentrated among older adults and those with other clinical conditions that increase the risk for severe COVID-19, as observed across other populations and time periods^1,2,5,6,10,11,19,20^. However, although we observed a slight narrowing of disparities during more recent time periods, a treatment gap existed across the quintiles of NDI among treatment eligible patients over the study period. These observed community-level social disparities persisted over time despite adjustment for individual-level clinical and demographic factors such as comorbidities or race/ethnicity, even during periods when treatment was widely available. Although most prior studies have not adequately controlled for these factors, similar patterns have been observed elsewhere^9,19,21,22^, including the apparent narrowing treatment gap between areas of differing vulnerability during more recent time periods^23^. Importantly, although health inequalities by socio-demographic characteristics pre-date the pandemic, efforts are needed to ensure that pre-existing health inequities are not exacerbated by the pandemic^24,25^. Indeed, general disparities in healthcare resources have been observed across many aspects of COVID-19 related care, from testing resources and positivity rates to vaccination rates and severe outcomes, even in settings without consumer healthcare costs^21,26^. The current analysis further suggests that the observed associations between demographic characteristics and treatment rates are unrelated to treatment costs since oral antivirals are provided free of charge within KPSC’s pre-paid healthcare plans. Hence, treatment disparities within other US healthcare systems may be significantly greater where there are associated costs such as prescription co-payments and consultation fees.

The results from our study suggest that patient awareness could play a role in the observed treatment disparities. Consistent with other studies conducted among highly vaccinated populations^2,27,28^, COVID-19 vaccination status (particularly ≥ 3 doses) was associated with higher nirmatrelvir–ritonavir uptake despite initial guidelines encouraging treatment prioritization among unvaccinated persons. Importantly, vaccination status is a known proxy for general healthcare-seeking behavior and risk perception, particularly in the context of COVID-19^29–32^. However, we also identified a potential lack of provider awareness in some cases, whereby some treatment eligible patients included in the chart review were not offered treatment because they were not perceived to meet the criteria for treatment. Available therapeutics and their corresponding guidelines changed rapidly throughout the pandemic^33^, potentially introducing confusion or hesitation regarding treatment decisions which could have contributed to misclassifications of treatment eligibility.

Importantly, although physician chart reviews were limited in this study, they provided some additional context surrounding the complexity of individual treatment decisions which previous studies have not assessed^19^. Specifically, the decision not to initiate treatment with nirmatrelvir–ritonavir among treatment eligible patients may have been clinically appropriate in some cases. For example, for some patients included in the chart review, consultations occurred too late following symptom onset for a perceived benefit of treatment, or symptoms had subsided by the time of the consultation. However, this delay in seeking healthcare did not appear to explain treatment allocation at the population level among treatment eligible patients, for whom the distribution of time from symptom onset to SARS-CoV-2 test date was similar between those who received nirmatrelvir–ritonavir compared with those who did not (Appendix L). Sometimes treatment decisions were further complicated by potential drug interactions according to some chart reviews, which were identified and discussed with the prescriber, leading to patients voluntarily deciding not to initiate treatment rather than withholding their medication for a pre-existing illness. Indeed, the NIH guidelines encourage prescribers to use their own clinical judgement to weigh individual benefits against potential risks on a case-by-case basis, particularly for patients on contraindicated medications or with pre-existing health conditions^7^.

As well as informing strategies for improved treatment access, our findings can assist with the design and interpretation of post-licensure nirmatrelvir–ritonavir effectiveness data, specifically when considering differences between nirmatrelvir–ritonavir recipients vs. non-recipients. For example, as well as demonstrating a clear difference in clinical risk between treatment groups (even within a treatment eligible cohort), the findings also show differences in socio-economic characteristics and factors related to patient awareness and general healthcare seeking behaviors between nirmatrelvir–ritonavir recipients and non-recipients, underscoring the need to account for these factors in future effectiveness studies. Inadequate adjustment may offer a partial explanation behind the heterogeneous effectiveness estimates observed across prior real-world population studies^1,2,4,5,11,20,34^. Reinforcing this concept, studies that were able to control for healthcare seeking behavioral factors (including delays to seeking care) and socio-economic characteristics in their analyses have produced effectiveness estimates closer to those produced by randomized controlled trials^4^.

Additionally, a relaxation in prescribing practices has eliminated the need for a laboratory-confirmed SARS-CoV-2 test, instead allowing patients to self-report antigen test results. As observed in the current study, due to the rise in availability of at-home tests, an increasing proportion of nirmatrelvir–ritonavir recipients do not have EHR-documented SARS-CoV-2 infection. This trend will make it more difficult to conduct population-representative real-world studies, as an increasing proportion of tests are likely to be conducted at home and not reported over time as COVID-19 public health emergencies end and pre-pandemic lifestyles resume.

### Limitations

Although the current study benefits from the availability of a large, rich dataset that represents encounters across all care settings, there are at least five limitations that should be considered when interpreting our findings. First, as mentioned previously, the primary analysis only included patients with documented evidence of SARS-CoV-2 in the KPSC EHR. Given this population sought care for a test, it is possible these patients had more severe illness compared to those who tested at home or did not report their test results. This potential selection bias could lead to overestimation of the true treatment rate if patients who seek care for testing are also more likely to seek treatment. Thus, treatment rates could be even lower than we and other studies that rely on documented evidence of SARS-CoV-2 observed^1,2,5,6,10,11^. In sensitivity analyses, however, we observed that there were very few differences in nirmatrelvir–ritonavir dispensing patterns between those with and without documented evidence of SARS-CoV-2 (Appendix I), indicating that observed disparities in treatment patterns likely persist regardless of the setting in which testing occurs. A second limitation is that while the KPSC population is diverse, our findings may not be generalizable to other populations or settings. For example, although KPSC communications regarding COVID-19 treatment guidelines closely mirrored NIH guidelines, they may have differed from guidance received by other health systems or prescribers. Third, our analysis did not account for nirmatrelvir–ritonavir supply constraints or changing guidelines over time, which may have explained some of the observed demographic or temporal variations in treatment allocation. However, our main analysis was stratified by time, and therefore the later periods should have been unaffected by changes to supply or guidelines. Fourth, many of the CDC-defined high-risk conditions are not adequately documented in EHR, such as ‘Wheelchair use’, and as mentioned above, treatment guidelines encouraged prescribers to apply their clinical judgement on a case-by-case basis. Therefore, although the ability to identify a treatment eligible cohort was a particular strength of the current study, it is likely that treatment eligible participants were underrepresented in this analysis. This could have biased the results if treatment eligibility criteria were less accurately documented within subgroups of the population. However, in sensitivity analyses that included all persons with a documented SARS-CoV-2 test regardless of treatment eligibility, nirmatrelvir–ritonavir dispensing patterns were similar across clinical and demographic characteristics (Appendix G). Lastly, the current study was unable to identify all possible underlying reasons for treatment decisions, and therefore it remains unclear whether population-level treatment gaps were mostly provider- or patient-led.

## Conclusion

This study represents a detailed analysis of nirmatrelvir–ritonavir dispensing patterns across COVID-19 patient characteristics using real-world data from a large and diverse US population. The proportion of treatment-eligible patients with SARS-CoV-2 infections receiving nirmatrelvir–ritonavir increased rapidly over time and, in general, aligned with tiered treatment guidelines. However, despite wider treatment availability and increased uptake over time, still more than 75% of treatment-eligible patients did not receive nirmatrelvir–ritonavir by the end of 2022. Furthermore, disparities were observed across socio-economic characteristics even after controlling for demographic and clinical characteristics. Future strategies aimed at enhancing patient and provider awareness are needed to improve utilization of this potentially life-saving antiviral.

### Supplementary Information

Supplementary Information.

## Data Availability

The datasets generated analyzed in the current study are not publicly available to protect patient confidentiality, but anonymized data might be made available by the investigative team if the inquirers agree to collaborate with the study team on all publications, provide external funding for the administrative and investigator time necessary for this collaboration, show that they are qualified and have documented evidence of training for human participant protections, and agree to abide by the terms outlined in data-use agreements between institutions. To request data, inquirers should contact the corresponding author.
