# Supplementary material for: Predictors of nirmatrelvir–ritonavir receipt among COVID-19 patients in a large US health system
Source: Sci Rep. 2024 Mar 29;14:7485. doi: 10.1038/s41598-024-57633-7 (PMC10980791; doi:10.1038/s41598-024-57633-7)
Supplement: Supplementary file 1 — Supplementary Information. [file 41598_2024_57633_MOESM1_ESM.pdf]

Appendix A. High-risk conditions for severe COVID-19, as defined by CDC

**Medical condition**
Alcoholic liver disease
Asthma
Attention-Deficit/Hyperactivity Disorder
Autism
Autoimmune hepatitis
Barthel Index (Disability Indicated by)
Bronchiectasis
Cancer
Cardiomyopathies
Cerebral Palsy
Cerebrovascular disease
Charcot Foot
Chromosomal Disorders
Chronic kidney disease
Chronic obstructive pulmonary disease
Cirrhosis
Cognitive Impairment
Congenital Hydrocephalus
Congenital Malformations
Cystic fibrosis
Deafness/Hearing Loss
Diabetes mellitus, type 1 and type 2
Disability Indicated by Barthel Index
Down Syndrome
Fahr’s Syndrome
Gaucher Disease
Hand and Foot Disorders
Heart failure
Human immunodeficiency virus
Interstitial lung disease
Learning disabilities
Leigh Syndrome
Leber's Hereditary Optic Neuropathy (LHON) or Autosomal Dominant Optic Atrophy (ADOA)
Maternal Inherited Diabetes and Deafness
Mobility Disability
Mood disorders, including depression
Movement Disorders
Multiple Disability (referred to in research papers as “bedridden disability”)
Multisystem Disease
Mitochondrial Encephalopathy, Lactic Acidosis, and Stroke-Like Episodes (MELAS) and Risk Markers
Myoclonic Epilepsy with Ragged Red Fibers (MERRF)
Myotonic Dystrophy
Neuromuscular Disorders
Neurodevelopmental Disorders
Neuromyelitis Optica Spectrum Disorder (NMOSD)
Neuropathy, Ataxia, and Retinitis Pigmentosa (NARP)
Neurologic conditions limited to dementia
Non-alcoholic fatty liver disease
Perinatal Spastic Hemiparesis
Primary Immunodeficiencies
Primary Mitochondrial Myopathy (PMM)
Progressive Supranuclear Palsy
Pulmonary embolism
Pulmonary hypertension
Schizophrenia spectrum disorders
Senior-Loken Syndrome
Solid organ or hematopoietic cell transplantation
Spina Bifida and Other Nervous System Anomalies
Spinal Cord Injury
Tourette Syndrome
Traumatic Brain Injury
Tuberculosis
Visual Impairment/Blindness

Appendix B. Medications that contraindicate the use of Nirmatrelvir/Ritonavir due to potential drug interactions

Drug/contraindication	Date implemented
Alfuzosin	31-Dec-21
Amiodarone	31-Dec-21
Carbamazepine	31-Dec-21
Dihydroergotamine	31-Dec-21
Eletriptan	28-Jun-22
Eplerenone	28-Jun-22
Finerenone	28-Jun-22
Flibanserin	28-Jun-22
Ivabradine	28-Jun-22
Lomitapide	28-Jun-22
Lumacaftor plus ivacaftor	28-Jun-22
Lurasidone	31-Dec-21
Methylergonovine	31-Dec-21
Midazolam (oral)	8-Apr-22
Maloxegol	28-Jun-22
Phenobarbital	31-Dec-21
Phenytoin	31-Dec-21
Pimozide	31-Dec-21
Primidone	28-Jun-22
Propafenone	31-Dec-21
Quinidine	31-Dec-21
Ranolazine	31-Dec-21
Rifampin	31-Dec-21
Sildenafil (Revatio®) when used for pulmonary arterial hypertension	31-Dec-21
Silodosin	28-Jun-22
Tolvaptan	28-Jun-22
Ubrogepant	28-Jun-22
Voclosporin	28-Jun-22
St John's Wort [Hypericum perforatum]	31-Dec-21

Appendix C. Diagnosis codes used to define chronic comorbidities

Comorbidity category	ICD-10 codes & definitions
**Diabetes**	
Type II diabetes mellitus	E11*: Type II diabetes mellitus
**Stroke**	
Stroke	I63*: Cerebral infarction.<br>I64*: Stroke, not specified as haemorrhage or infarction<br>I69*: Sequelae of cerebrovascular disease<br>G45*: Transient cerebral ischaemic attacks and related syndromes<br>G46*: Vascular syndromes of brain in cerebrovascular diseases
Stroke - hemorrhagic	I60*: Nontraumatic subarachnoid hemorrhage<br>I61*: Nontraumatic intracerebral hemorrhage<br>I62*: Other and unspecified nontraumatic intracranial hemorrhage
**Coronary Heart Disease**	
Acute myocardial infarction	I21.0: Acute transmural myocardial infarction of anterior wall<br>I21.1: Acute transmural myocardial infarction of inferior wall<br>I21.2: Acute transmural myocardial infarction of other sites<br>I21.3: Acute transmural myocardial infarction of unspecified site<br>I21.4: Acute subendocardial myocardial infarction<br>I21.9: Acute myocardial infarction, unspecified
Subsequent MI	I22.0: Subsequent myocardial infarction of anterior wall<br>I22.1: Subsequent myocardial infarction of inferior wall<br>I22.8: Subsequent myocardial infarction of other sites<br>I22.9: Subsequent myocardial infarction of unspecified site
Complications following MI	I23.0: Haemopericardium as current complication following acute myocardial infarction;<br>I23.1:Atrial septal defect as current complication following acute myocardial infarction;<br>I23.2: Ventricular septal defect as current complication following acute myocardial infarction;<br>I23.3: Rupture of cardiac wall without haemopericardium as current complication following acute myocardial infarction;<br>I23.4: Rupture of chordae tendineae as current complication following acute myocardial infarction;<br>I23.5:Rupture of papillary muscle as current complication following acute myocardial infarction;<br>I23.6: Thrombosis of atrium, auricular appendage, and ventricle as current complications following acute myocardial infarction;<br>I23.8: Other current complications following acute myocardial infarction
Coronary Heart Disease	I24.9: Acute ischaemic heart disease, unspecified (excl. ischaemic heart disease (chronic) NOS)<br>I25.6: Silent myocardial ischaemia<br>I25.8: Other forms of chronic ischaemic heart disease specified as chronic<br>I25.9: Chronic ischaemic heart disease, unspecified -Ischaemic heart disease (chronic) NOS
**Chronic kidney disease**	
Chronic kidney disease	N18*: Chronic kidney disease
Other CKD	E08.22: Diabetic chronic kidney disease<br>N19*: Unspecified kidney failure
**Heart Failure**	
Heart failure	I50.0: Congestive heart failure<br>I50.1: Left ventricular failure<br>I50.9: Heart failure, unspecified
Ischemic cardiomyopathy	I42.0: Dilated cardiomyopathy<br>I42.9: Cardiomyopathy, unspecified
Hypertensive heart failure	I11.0: Hypertensive heart disease with congestive heart failure
Ischemic cardiomyopathy	I25.5: Ischemic cardiomyopathy
Hypertensive heart failure	I13.2: Hypertensive heart and renal disease with heart failure and renal failure<br>I13.0: Hypertensive heart and renal disease with (congestive) heart failure
**COPD**	
COPD	J44.9: Chronic Obstructive Pulmonary Disease [COPD]<br>J44.0: Chronic obstructive pulmonary disease [COPD] with acute bronchitis

COPD = Chronic Obstructive Pulmonary Disease; CKD = Chronic Kidney Disease; ICD-10 = International Classification of Diseases 10th revision; MI= Myocardial infarction;

Appendix D**.** Study population flow chart

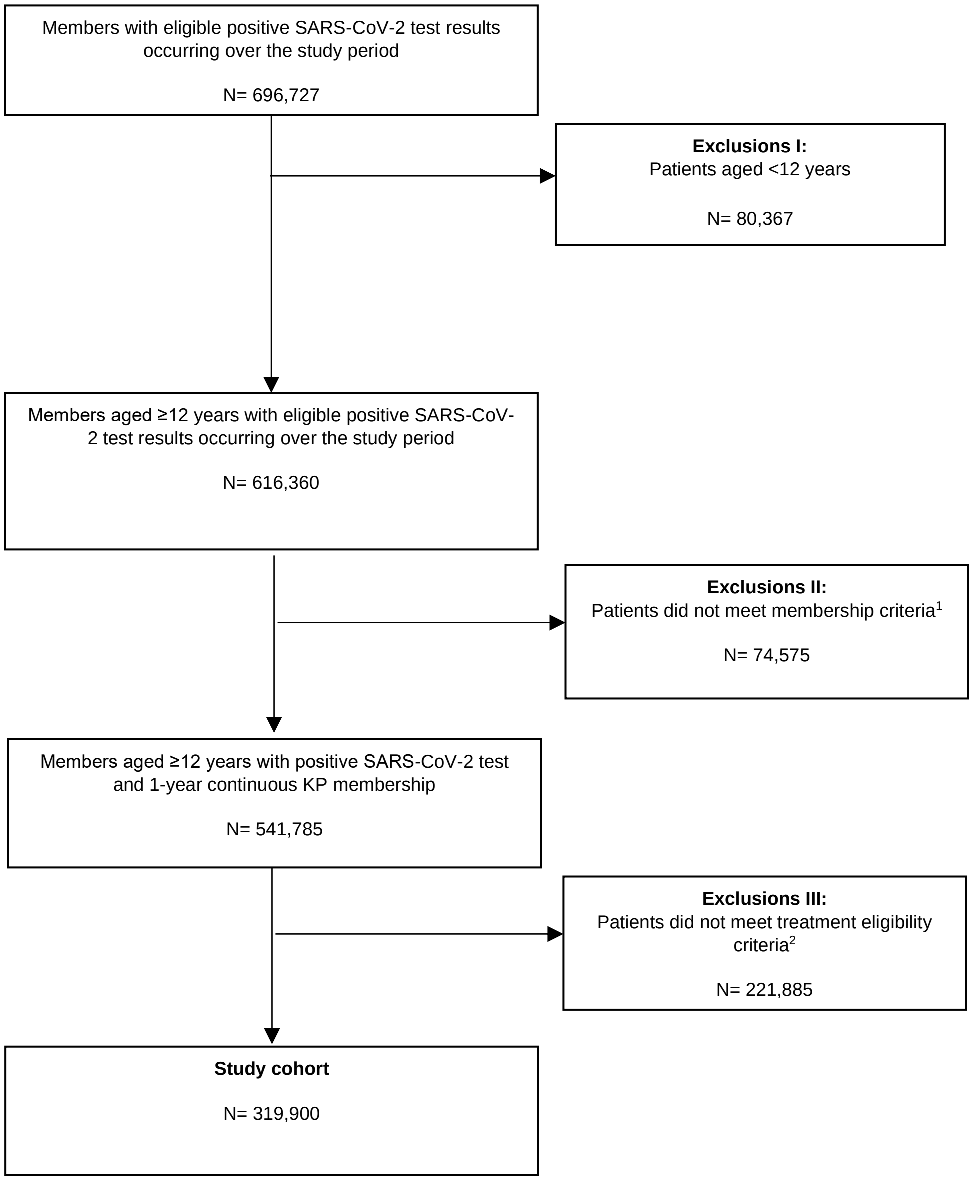

[1] Full continuous healthcare membership 1 year prior, allowing a 45 day enrollment gap.
[2] Treatment eligibility was determined according to the US National Institutes of Health (NIH) COVID-19 Treatment Guidelines for Nirmatrelvir/Ritonavir.
Eligible positive tests included EHR documentation of a positive SARS-CoV-2 tests (antigen [including self-reported] and PCR)

Appendix E**.** Number of patients with SARS-CoV-2 infection identified as treatment eligible according to each treatment eligibility criteria

**Eligibility criteria***	**N**	**Percentage (%) of treatment-eligible patients**
Age 65+	63,921	20.0
Current/Former smoker	80,536	25.2
High risk conditions	200,793	62.8
Immunocompromised	87,291	27.3
Pregnant within the past year	11,606	3.6

*Categories are not mutually exclusive

Appendix F. Odds Ratio (95% CI) of nirmatrelvir-ritonavir treatment dispense among SARS-CoV-2 positive patients across NDI (top vs. bottom third of NDI) with at least one qualifying treatment eligible characteristic, stratified by time period of infection, age and race/ethnicity

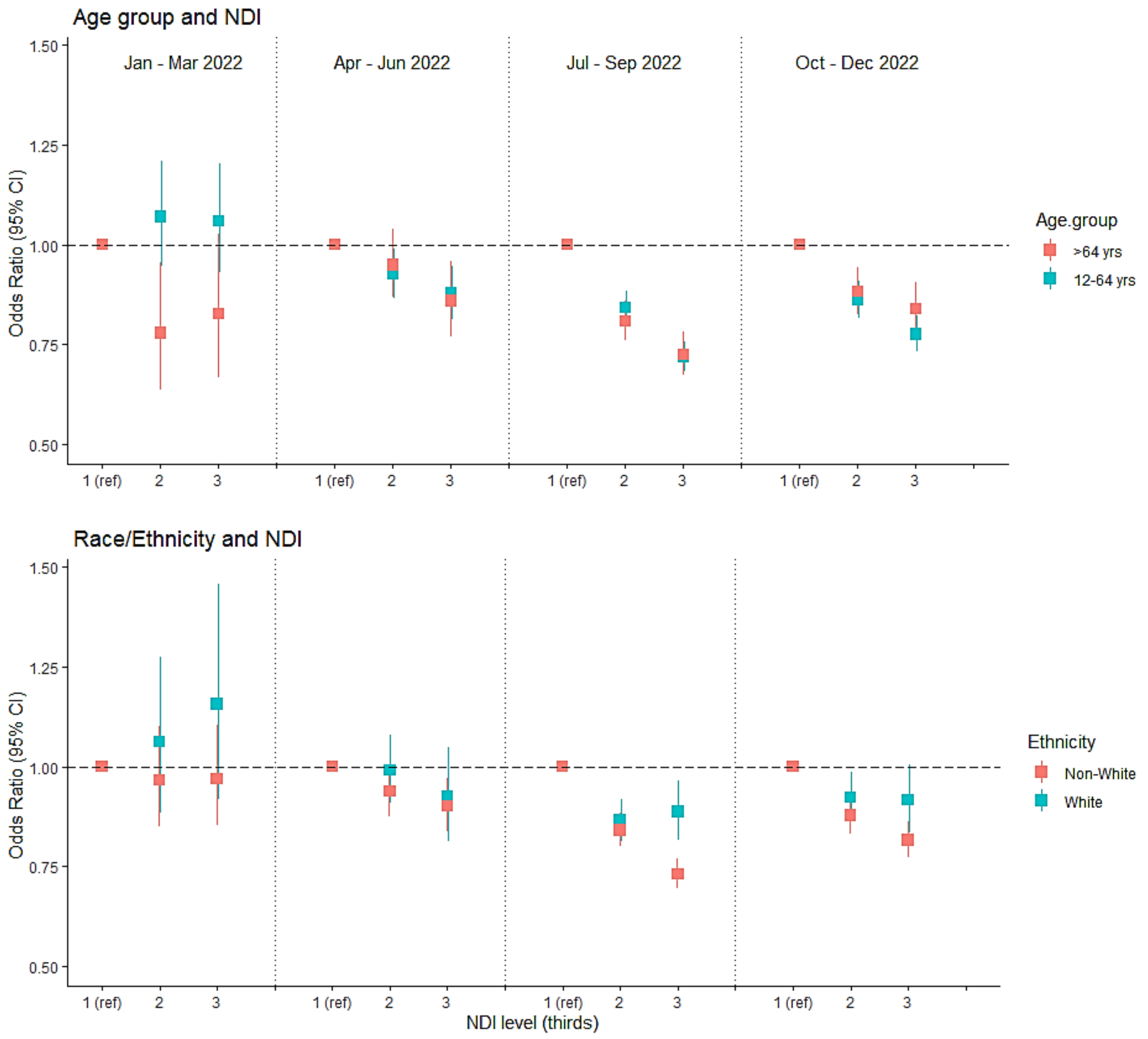

NDI = Neighborhood deprivation index (stratified by equal thirds of the study population)

Appendix G. Proportion of all patients with SARS-CoV-2 infection over the study period receiving nirmatrelvir-ritonavir, by selected baseline characteristics

	All documented SARS-CoV-2 infections	
	**Total, col %**	**Treatment dispensed, row %**
**Total**	541,785	44,645 (8.2%)
**Sex**		
Men	233227 (43.0%)	18635 (8.0%)
Women	308558 (57.0%)	26010 (8.4%)
**Age, years**		
12-17	34696 (6.4%)	186 (0.5%)
18-30	86855 (16.0%)	1896 (2.2%)
31-44	149869 (27.7%)	7061 (4.7%)
45-64	182270 (33.6%)	17553 (9.6%)
65+	88095 (16.3%)	17949 (20.4%)
Mean (SD)	45.4 (18.1)	58.6 (15.9)
**Race/Ethnicity**		
Asian	69366 (12.8%)	6519 (9.4%)
Black	41346 (7.6%)	3267 (7.9%)
Hispanic	249838 (46.1%)	16144 (6.5%)
Other/Unknown	32910 (6.1%)	1730 (5.3%)
White	148325 (27.4%)	16985 (11.5%)
**BMI, kg/m^2***		
<18.5	11493 (2.1%)	401 (3.5%)
18.5-24.9	129550 (23.9%)	9144 (7.1%)
25-29.9	166537 (30.7%)	14003 (8.4%)
30+	219587 (40.5%)	20801 (9.5%)
Unknown	14618 (2.7%)	296 (2.0%)
**Insurance plan**		
Medicaid	52673 (9.7%)	3703 (7%)
Medicare	71450 (13.2%)	15000 (21%)
Commercial	389311 (71.9%)	23787 (6.1%)
Other	26291 (4.9%)	2022 (7.7%)
Unknown	2060 (0.4%)	133 (6.5%)
**NDI, quintiles**		
Q1	113586 (21.0%)	12216 (10.8%)
Q2	117008 (21.6%)	10429 (8.9%)
Q3	112874 (20.8%)	8731 (7.7%)
Q4	106647 (19.7%)	7513 (7%)
Q5	91557 (16.9%)	5745 (6.3%)
**Unknown**	113 (0.0%)	11 (9.7%)
**High-risk comorbidities*┼**		
0-1	464570 (85.7%)	30846 (6.6%)
2-3	47687 (8.8%)	8840 (18.5%)
4+	29528 (5.5%)	4959 (16.8%)
**Specific comorbidities***		
Chronic kidney disease	24285 (4.5%)	3677 (15.1%)
Coronary heart disease	2895 (0.5%)	365 (12.6%)
Diabetes (Type II)	73286 (13.5%)	11514 (15.7%)
Heart failure	9550 (1.8%)	1277 (13.4%)
Stroke	5090 (0.9%)	741 (14.6%)
COPD	6795 (1.3%)	1335 (19.6%)

* Documented within the year prior to positive SARS-CoV-2 test.
┼ High-risk co-morbidities defined according to ICD-10 codes included in **Appendix A**
NDI = Neighborhood deprivation index; BMI = Body-Mass Index

Appendix H. Types of diagnosis test used to identify SARS-CoV-2 infection, by nirmatrelvir-ritonavir dispense

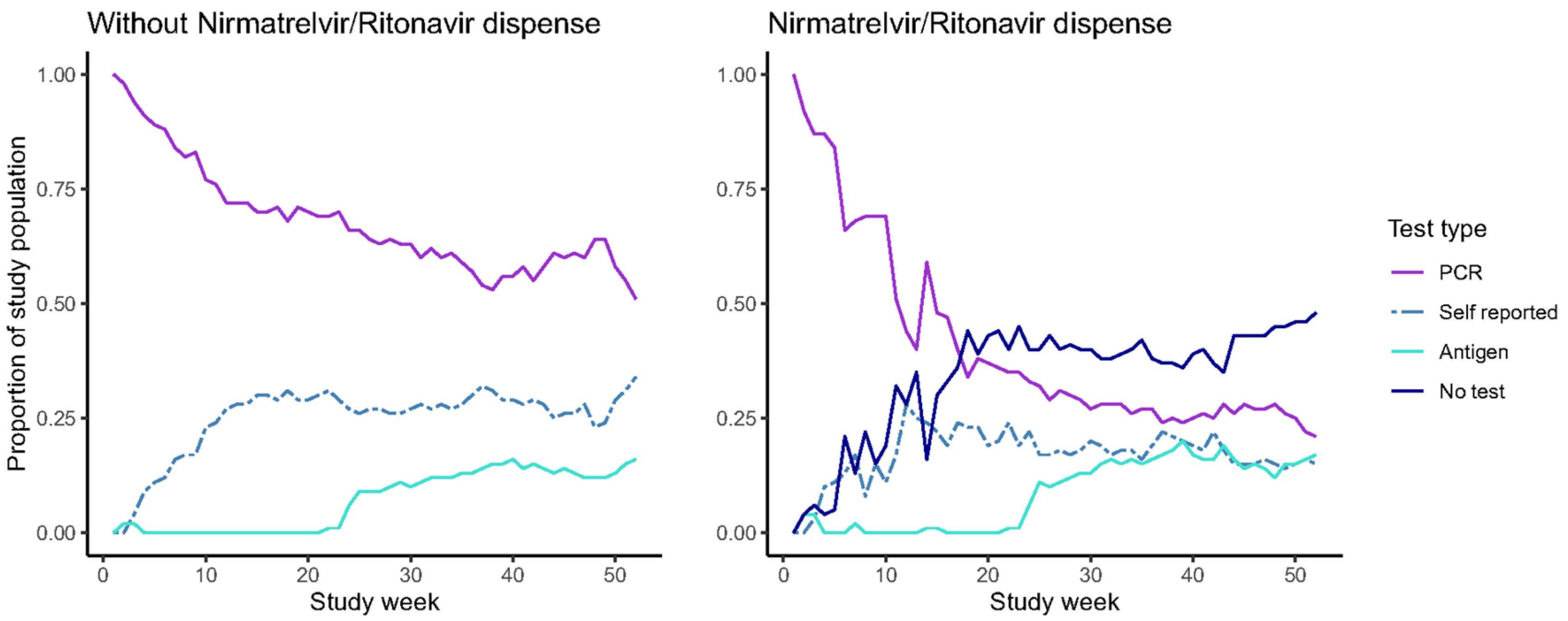

Appendix I. Characteristics of patients that received a prescription for nirmatrelvir-ritonavir without a documented SARS-CoV-2 infection

	Nirmatrelvir-ritonavir dispenses, N			Percent (%) of nirmatrelvir-ritonavir dispenses without a documented SARS-CoV-2 infection
	**All**	**Documented SARS-CoV-2 infection**	**No documented SARS-CoV-2 infection**	
**All**	75196	44645	30551	41%
**Age at dispense**				
12-17	374	186	188	50%
18-30	3161	1896	1265	40%
31-44	10738	7061	3677	34%
45-64	28973	17553	11420	39%
65+	31950	17949	14001	44%
**Sex**				
Women	31710	18635	13075	41%
Men	43486	26010	17476	40%
**Race/ethnicity**				
Asian	11202	6519	4683	42%
Black	5007	3267	1740	35%
Hispanic	27384	16144	11240	41%
Other/Unknown	3047	1730	1317	43%
White	28556	16985	11571	41%
**COVID-19 vaccine dose count**				
0	4774	2812	1962	41%
1	1284	767	517	40%
2	10653	6286	4367	41%
3+	58485	34780	23705	41%
**NDI (quintiles)**				
Q1	20642	12216	8426	41%
Q2	17520	10429	7091	40%
Q3	14675	8731	5944	41%
Q4	12786	7513	5273	41%
Q5	9554	5745	3809	40%
**Chronic comorbidities**				
Chronic kidney disease	6426	3677	2749	43%
Coronary heart disease	675	365	310	46%
Diabetes (Type II)	19502	11514	7988	41%
Heart failure	2142	1277	865	40%
Stroke	1325	741	584	44%
COPD	2279	1335	944	41%
**Prior SARS-CoV-2 infection**				
Yes	6552	3951	2601	40%
No	68644	40694	27950	41%
**At least one healthcare encounter in the year prior**				
Outpatient visit	73172	43553	29619	40%
Inpatient/ED visit	18685	11376	7309	39%
Telehealth visit	58588	34922	23666	40%
No encounters	1286	725	561	44%

Appendix J. Characteristics of patients that received a prescription for nirmatrelvir-ritonavir, by dispense status

	**Prescribed, N (%)**	**Prescribed but not dispensed, N (%)**	**Percent (%) of nirmatrelvir-ritonavir prescriptions without dispense**
**All**	44610 (100%)	7838 (100%)	14.9%
**Age at prescription or dispense**			
12-17	181 (0%)	62 (1%)	25.5%
18-30	1879 (4%)	595 (8%)	24.1%
31-44	7039 (16%)	1826 (23%)	20.6%
45-64	17532 (39%)	3332 (43%)	16.0%
65+	17979 (40%)	2023 (26%)	10.1%
**Sex**			
Women	25987 (58%)	4769 (61%)	15.5%
Men	18623 (42%)	3069 (39%)	14.1%
**Race/ethnicity**			
Asian	6521 (15%)	968 (12%)	12.9%
Black	3271 (7%)	565 (7%)	14.7%
Hispanic	16137 (36%)	3123 (40%)	16.2%
Other/Unknown	1703 (4%)	377 (5%)	18.1%
White	16978 (38%)	2805 (36%)	14.2%
**COVID-19 vaccine dose count**			
0	2810 (6%)	857 (11%)	23.4%
1	763 (2%)	207 (3%)	21.3%
2	6268 (14%)	1494 (19%)	19.2%
3+	34769 (78%)	5280 (67%)	13.2%
**NDI (quintiles)**			
Q1	12208 (27%)	2032 (26%)	14.3%
Q2	10420 (23%)	1779 (23%)	14.6%
Q3	8727 (20%)	1552 (20%)	15.1%
Q4	7508 (17%)	1422 (18%)	15.9%
Q5	5736 (13%)	1050 (13%)	15.5%
**Weighted Charlson Comorbidities**			
0-1	30818 (69%)	6028 (77%)	16.4%
2-3	8835 (20%)	1140 (15%)	11.4%
4+	4957 (11%)	670 (9%)	11.9%
**Time of symptom onset relative to SARS-CoV-2 test**			
After test date	1082 (2%)	208 (3%)	16.1%
Before test date	36965 (83%)	6435 (82%)	14.8%
Same date	5891 (13%)	1064 (14%)	15.3%
No symptoms	672 (2%)	131 (2%)	16.3%
**Treatment eligible**			
Yes	34769 (78%)	5977 (76%)	14.7%
No	9841 (22%)	1861 (24%)	15.9%
**At least one healthcare encounter in the year prior**			
Outpatient visit	43520 (98%)	7532 (96%)	14.8%
Inpatient/ED visit	11363 (25%)	1900 (24%)	14.3%
Telehealth visit	34896 (78%)	6113 (78%)	14.9%
No encounters	724 (2%)	195 (2%)	21.2%

Appendix K. Medical chart review findings among 40 patients with no dispense but identified as eligible for treatment according to EHR

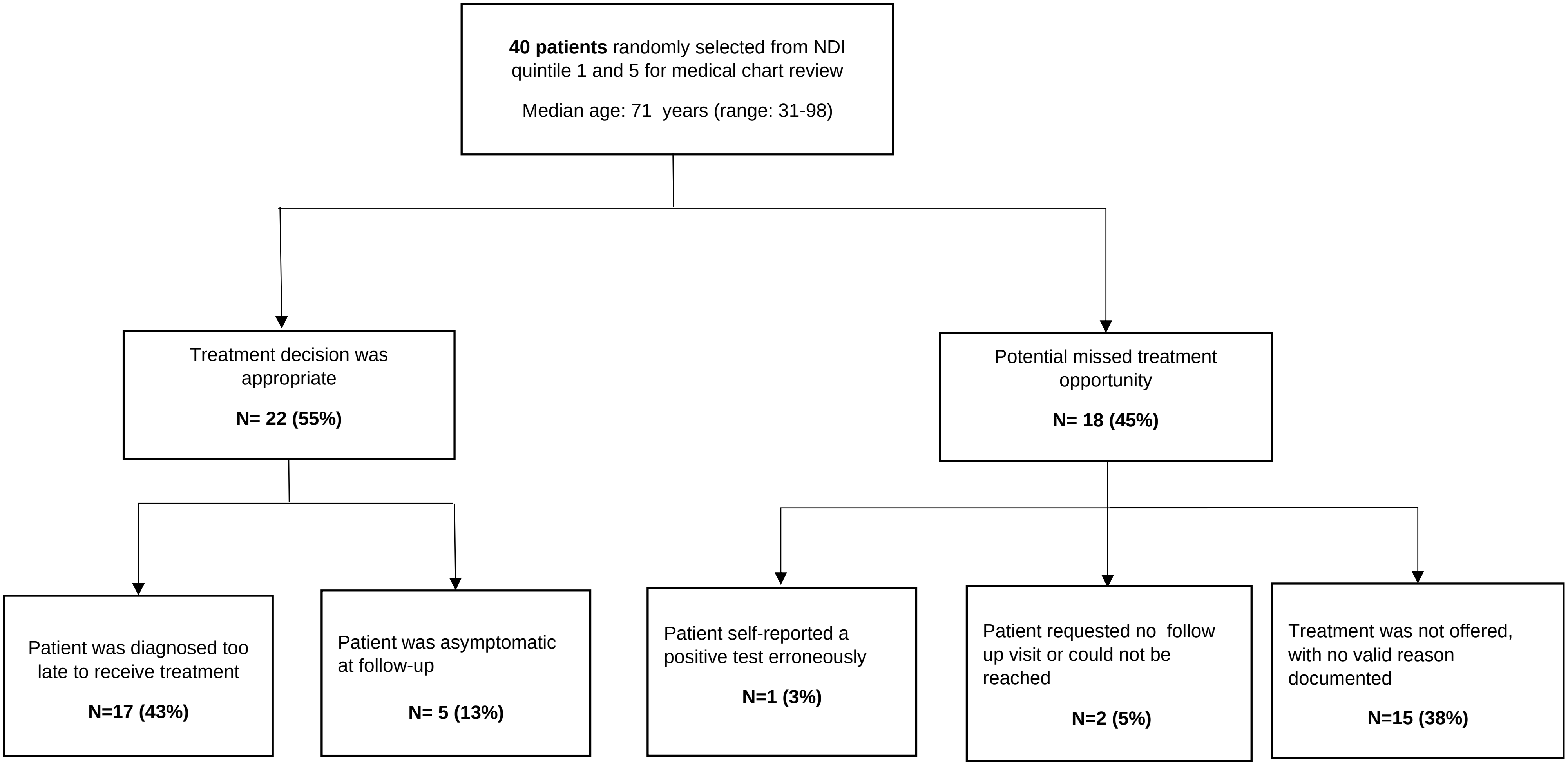

NDI = Neighborhood deprivation index

Appendix L. Distribution of days from symptom onset to SARS-CoV-2 test among patients identified as treatment eligible*, by nirmatrelvir-ritonavir dispense status

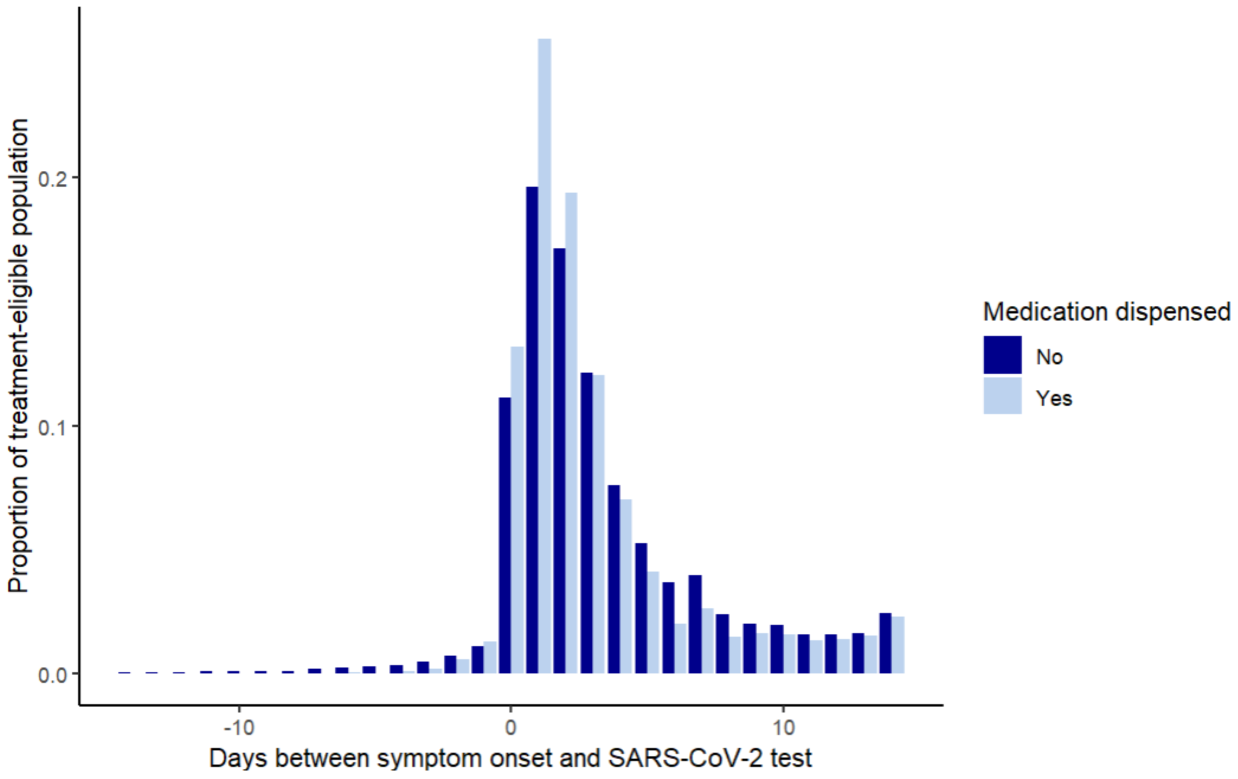

* Treatment eligibility was determined according to the US National Institutes of Health (NIH) COVID-19 Treatment Guidelines for Nirmatrelvir/Ritonavir
